# Chronic opioid pretreatment potentiates the sensitization of fear learning by trauma

**DOI:** 10.1038/s41386-019-0559-5

**Published:** 2019-12-02

**Authors:** Zachary T. Pennington, Jeremy M. Trott, Abha K. Rajbhandari, Kevin Li, Wendy M. Walwyn, Christopher J. Evans, Michael S. Fanselow

**Affiliations:** 1 Department of Psychology, University of California, Los Angeles, CA USA; 2 Staglin Center for Brain and Behavioral Health, University of California, Los Angeles, CA USA; 3 Department of Neuroscience, Mount Sinai School of Medicine, New York, NY USA; 4 Department of Psychiatry and Biobehavioral Sciences, University of California, Los Angeles, CA USA; 5 Hatos Center for Neuropharmacology, University of California, Los Angeles, CA USA

**Keywords:** Psychology, Neuroscience

## Abstract

Despite the large comorbidity between PTSD and opioid use disorders, as well as the common treatment of physical injuries resulting from trauma with opioids, the ability of opioid treatments to subsequently modify PTSD-related behavior has not been well studied. Using the stress-enhanced fear learning (SEFL) model for PTSD, we characterized the impact of chronic opioid regimens on the sensitization of fear learning seen following traumatic stress in mice. We demonstrate for the first time that chronic opioid pretreatment is able to robustly augment associative fear learning. Highlighting aversive learning as the cognitive process mediating this behavioral outcome, these changes were observed after a considerable period of drug cessation, generalized to learning about multiple aversive stimuli, were not due to changes in stimulus sensitivity or basal anxiety, and correlated with a marker of synaptic plasticity within the basolateral amygdala. Additionally, these changes were not observed when opioids were given after the traumatic event. Moreover, we found that neither reducing the frequency of opioid administration nor bidirectional manipulation of acute withdrawal impacted the subsequent enhancement in fear learning seen. Given the fundamental role of associative fear learning in the generation and progression of PTSD, these findings are of direct translational relevance to the comorbidity between opioid dependence and PTSD, and they are also pertinent to the use of opioids for treating pain resulting from traumas involving physical injuries.

## Introduction

Post-traumatic stress disorder (PTSD) is highly comorbid with substance use disorder (SUD), with nearly 40% of individuals with PTSD also having SUD [1–6]. Understanding this comorbidity could shed fundamental mechanistic light on disease origins. Traditional models of PTSD–SUD comorbidity propose that SUD emerges as a consequence of PTSD. In support of this, longitudinal studies confirm that PTSD increases risk for SUD [4] and animal studies have shown that stressors are able to potently drive drug-seeking [7]. Nevertheless, this does not preclude the possibility that substance use is also able to influence the development of PTSD. The potential ability of opioid use to augment PTSD is particularly relevant to PTSD–SUD comorbidity. Large-scale epidemiological studies indicate that nearly 35% of individuals with an opioid use disorder have comorbid PTSD [3, 8, 9], among the highest of any SUD [3]. This raises the concern that opioid use could directly facilitate PTSD development. Moreover, because individuals that experience trauma are commonly prescribed opioids, the ability of these medications to alter the progression of PTSD is an important factor to consider. Here, we assessed the impact of chronic opioid regimens on the subsequent development of PTSD-like behaviors using the stress-enhanced fear learning (SEFL) model. We have previously demonstrated that SEFL captures several lasting anxiogenic changes in response to traumatic stress, including augmented fear learning, increased anxiety and startle responses, and altered glucocorticoid cycling [10–14]. Moreover, this model has recently been used to characterize long-lasting trauma-induced changes in drug seeking [15], making it optimal to study bidirectional interactions between trauma and drug use. We found that opioid exposure was able to markedly potentiate the ability of trauma to augment fear learning, and that this persisted at least a week after opioid cessation, a time in which weight changes, anxiety-like differences and differences in shock reactivity, were not apparent. Moreover, this change appears to be a direct ramification of opioid exposure rather than acute withdrawal.

## Methods and materials

### Animals

C57 BL/6J mice, 2–3 months of age, were obtained from Jackson Laboratories and housed individually for 2–4 weeks before testing. After initially showing that the effects of morphine were the same in both sexes, the remaining experiments were performed in males. The Chancellor’s Animal Research Committee at UCLA approved all experiments.

### Drug treatments

#### Chronic morphine injections

Animals were administered a common escalating regimen of twice-daily morphine sulfate (National Institutes of Drug Abuse; Bethesda MD), or an equivalent volume of saline, over the course of eight consecutive days. The dose of each injection on successive days was as follows: Day 1 = 10 mg/kg; Day 2 = 20 mg/kg; Day 3 = 30 mg/kg; Day 4 = 40 mg/kg; Days 5–8 = 50 mg/kg. Injections were subcutaneously administered daily between 8–10 a.m. and 5–7 p.m at a volume of 10 ml/kg. This dosing regimen was intended to mimic a pattern of escalating and cyclical systemic dosing. When the rate of morphine injections was varied (1× vs. 2× daily) the number of injections remained constant (i.e. 16 injections were administered over 8 or 16 days) so that cumulative morphine exposure remained constant.

#### Morphine pellet implantation and repeated naltrexone-precipitated withdrawal

Morphine pellets containing 8.3 mg of morphine, wrapped in sterilized nylon, or placebo pellets, were subcutaneously implanted below the neck to provide a continuous supply of morphine. Pellets containing 25 mg morphine were obtained from NIDA and cut down to size and placebo pellets were treated similarly. In order to produce cycling of morphine withdrawal, mice were treated with naltrexone twice-daily (0.25 mg/kg, i.p.; mornings/evenings), for 7 days, beginning the day after pellet implantation. Withdrawal was assessed in the morning of days 1, 3, 5, and 7, post-implant. To do so, animals were placed in a translucent plexiglass cylinder (15.24 cm wide by 38.1 cm tall) for 15 min immediately after saline/naltrexone injection. The plexiglass cylinder was set atop a piece of clean absorbent cloth that was weighed before/after the session to assess excretion. Additionally, the number of jumps observed in the final 10 min of each session was counted.

### Behavioral testing

#### SEFL procedure

The SEFL procedure, which captures the ability of trauma to sensitize fear learning [13, 16], took place across 4 days. Prior to this, all animals were habituated to handling for 3 days, ~60 s/day, and were also habituated to transport from the vivarium to the laboratory for 2 days, 15 min/day. On the first day, animals experienced the traumatic stressor, consisting of 10, 1 mA, 1 s shocks, pseudo-randomly distributed over the course of an hour in a distinctly configured conditioning chamber/context. Non-trauma animals were placed in the context for an equivalent amount of time. Shock reactivity, a measure of nociception, was assessed by examining average motion during shock periods, and freezing throughout trauma was assessed during 30 s intervals, beginning 30 s after each shock. On the second day, animals were re-exposed to the context of the traumatic stressor for 8 min to assess their memory of the traumatic event. On the third day, animals were exposed to a mild stressor in a novel environment. After 3 min exploring the novel chamber/context, animals were given a 0.5 mA, 2 s, shock. They were taken out of the chamber 2 min later. On the fourth day, animals were placed back in the context of the mild stressor for 8 min. Detailed information on shock equipment can be found in the Supplemental Methods. Notably, although shock was used here for the mild stressor, we have also found SEFL using a loud auditory startle stimulus [17], demonstrating that this phenomenon is not specific to the aversive stimulus employed. An extensive demonstration that SEFL is a phenomenon of enhanced learning can also be found in Rau et al. [13].

Additional details regarding the method of assessing shock reactivity and for elevated plus maze (EPM) testing can be found in the Supplemental Methods.

### Tissue collection, immunohistochemistry, and cell counts

Detailed immunohistochemical protocols and image analysis can be found in the Supplemental Methods.

### Statistical analysis

Data were analyzed using the general linear model. Detailed information on statistical procedures can be found in the Supplemental Methods.

## Results

### Chronic opioid pretreatment potentiates SEFL

**Fig. 1:**
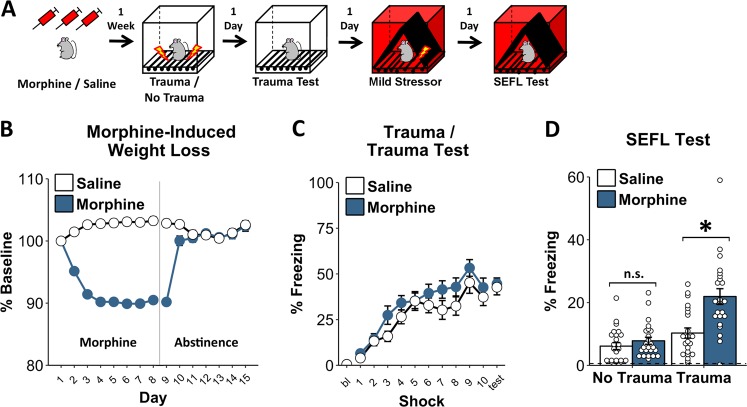
Chronic opioid pretreatment potentiates SEFL. **a** Schematic of the SEFL procedure. During the trauma, animals received 10, 1 s, 1 mA shocks. The next day they were returned for the trauma test. Subsequently, animals received a single 2 s, 0.5 mA shock, in a novel environment (the mild stressor). The following day they were placed back in this environment for the SEFL test. **b** By the end of the weeklong cessation period when they received the trauma, morphine-treated animals’ weight was no longer different from saline-treated animals. **c** Throughout the trauma and when subsequently returned to the trauma context for the trauma test, morphine- and saline-treated animals did not differ. **d** After being given a single shock in a novel environment, animals that underwent trauma froze more than non-trauma animals when returned to that environment—evidence of SEFL—and morphine-treated animals showed this effect to a much greater degree. Dashed line in **d** reflects average pre-shock baseline freezing on the previous day. Error bars reflect standard error of the mean. Asterisk reflects significance at *p* < 0.05. n.s. not significant, bl baseline. Group sizes for **b**–**d**: *n* = 22–24/group

The impact of chronic opioid exposure on fear learning was first assessed using the SEFL model for PTSD, which captures the sensitization of fear learning observed following traumatic experience (Fig. 1a), in addition to several other phenotypes relevant to PTSD [13, 14, 16]. Because prior trauma is a major predictor of who will develop PTSD in response to a subsequent traumatic event [1, 18] this phenomenon is highly relevant to understanding PTSD development.

Animals were treated across 8 days with twice-daily escalating doses of morphine (10–50 mg/kg), or saline, and were then given a week of drug cessation prior to being behaviorally tested using the SEFL procedure (Fig. 1a). Notably, by the time of behavioral testing, morphine-treated animals had recovered from the large morphine-induced weight loss observed (Fig. 1b. Morphine effect on Day 8: *F*_1,89_ = 321, *p* < 0.001; morphine effect on Day 15: *F*_1,89_ = 0.29, *p* = 0.59), suggesting that they were beyond the period of acute withdrawal.

During the initial trauma, animals received 10 footshocks across the course of an hour. We have called this the trauma because this acute stressor is able to produce a lasting sensitization of fear and anxiety-like behaviors [13, 16]. Importantly, throughout the trauma, morphine-treated animals did not show altered shock reactivity (*F*_1,41_ = 0.22, *p* = 0.65; data not shown) nor did they freeze more than saline-treated animals after each shock (Fig. 1c. Effect of morphine in trauma groups: *F*_1,41_ = 2.86, *p* = 0.1; morphine × trial interaction: *F*_9,369_ = 0.57, *p* = 0.75). Moreover, both groups reached an equivalent high level of freezing when they were returned to the trauma environment the next day for the trauma test (Fig. 1c. Effect of morphine in trauma groups: *F*_1,41_ = 0.11, *p* = 0.74). Thus, the initial response to trauma appeared to be unaltered by morphine pre-exposure.

Animals were next placed into a novel environment and given a single mild footshock (0.5 mA, 2 s). When first placed in this environment (i.e., prior to shock), traumatized animals did not display generalized fear relative to animals that had not experienced the trauma (indicated by the amount of freezing), and morphine- and saline-treated animals did not differ (effect of morphine: *F*_1,85_ = 1.7, *p* = 0.2; morphine × trauma interaction: *F*_1,85_ = 0.25, *p* = 0.62; mean baseline freezing in traumatized animals = 0.8%. Data not shown). Furthermore, when given the mild stressor, morphine- and saline-treated animals did not differ in their motor response to this stimulus (effect of morphine: *F*_1,85_ < 0.01, *p* = 0.97; morphine × trauma interaction: *F*_1,85_ = 0.01, *p* = 0.9. Data not shown). However, when returned to this environment the next day, morphine-treated animals displayed a profound sensitization of SEFL: animals that experienced the trauma froze more in the environment that had been paired with the mild stressor (Fig. 1d. Effect of trauma: *F*_1,85_ = 22.73, *p* < 0.001), and morphine-treated animals displayed this enhancement following trauma to a much greater degree than saline-treated animals (Fig. 1d. Morphine × trauma interaction: *F*_1,85_ = 8.61, *p* < 0.01). Although morphine increased freezing among animals that had received the trauma (*F*_1,41_ = 12.78, *p* < 0.001), morphine-treated animals that had not experienced the trauma did not display heightened fear levels relative to saline-treated animals (*F*_1,44_ = 0.42, *p* = 0.52). Notably, both males and females showed this effect to a similar degree (Fig. S1), supporting the robust nature of this phenomenon.

### Potentiation of SEFL by chronic opioid pretreatment is not due to changes in stimulus sensitivity or anxiety

**Fig. 2:**
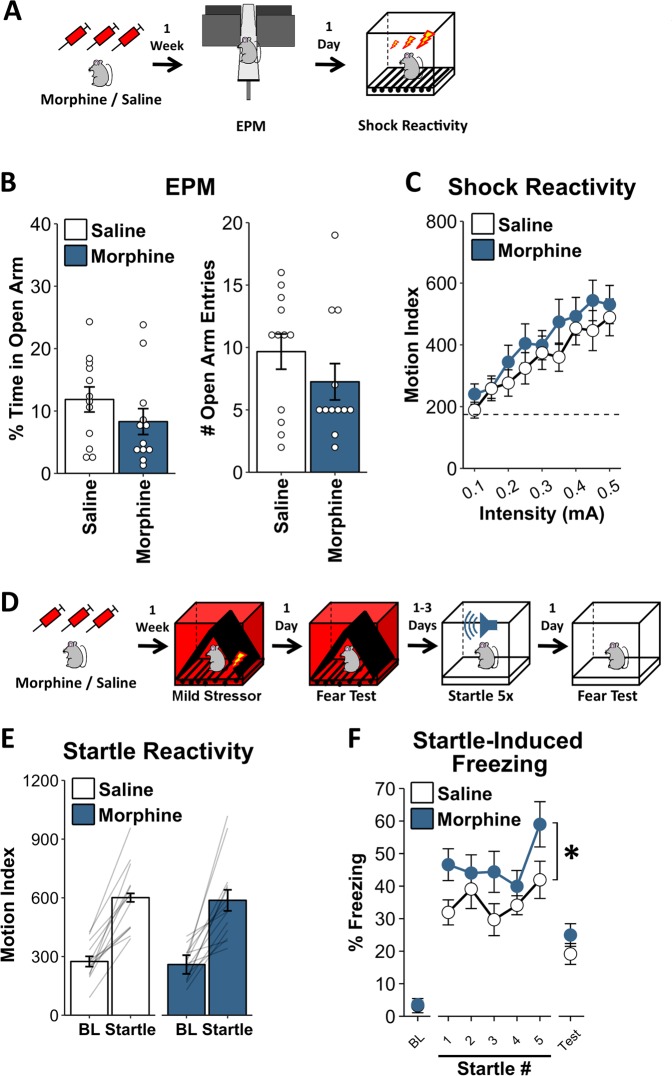
Chronic opioid pretreatment does not alter shock sensitivity or anxiety. **a** Experiment Schematic. A week after chronic morphine/saline exposure, animals were tested in the EPM. The next day, shock reactivity was assessed. **b** Morphine-treated animals did not display altered exploration of the open arms of the EPM. **c** Morphine-treated animals did not display altered shock reactivity. See Supplemental Methods for details. Dashed line in **c** reflects pre-shock motion prior to the first shock, which did not differ between groups (*t*_22_ = 0.1, *p* = 0.92). **d** Experiment Schematic. A subset of animals which received morphine/saline and were fear conditioned with a single 2 s, 0.5 mA shock, were subsequently exposed to a series of auditory startle stimuli (2 s, 115 db, white noise, each separated by 1 min) in a novel environment, and were placed back in the startle-paired environment the next day. **e** Morphine-treated animals did not differ with respect to baseline motion or startle-induced increases in motion. **f** Morphine-treated animals did not differ with respect to baseline freezing when initially placed in the startle-paired environment but showed increased post-startle freezing, a learned behavior. Nevertheless, when placed back into the startle-paired environment the next day, morphine-treated animals did not significantly differ from saline-treated animals. Error bars reflect standard error of the mean. BL baseline. For **a**–**c**, *n* = 12/group. For **d**–**f**, *n* = 13–14/group

In order to parse whether enhancements in fear were a consequence of altered learning processes, or some other factor influencing the expression of fear, we assessed the impact of morphine exposure on a measure of general anxiety as well as shock reactivity (Fig. 2a–c). We found that the morphine regimen previously used failed to alter anxiety-like behavior in the EPM at a time equivalent to when trauma was given (Fig. 2b. Percent time in open arms: *t*_22_ = 1.23, *p* = 0.23; open arm entries: *t*_22_ = 1.19, *p* = 0.25). Additionally, although shock reactivity increased with shock intensity, prior morphine treatment did not influence shock reactivity across a wide range of shock amplitudes (Fig. 2c. Effect of intensity: *F*_8,176_ = 22.81, *p* < 0.001; effect of morphine: *F*_1,22_ = 0.84, *p* = 0.37; morphine × intensity interaction: *F*_8,176_ = 0.68, *p* = 0.61).

Demonstrating that morphine’s impact on fear learning generalized to other aversive stimuli, morphine-treated animals also showed heightened freezing after exposing them to auditory startle stimuli (Fig. 2d–f). A subset of animals which received morphine/saline and were fear conditioned with a single 2 s, 0.5 mA shock, were subsequently exposed to a series of auditory startle stimuli (2 s, 115 db, white noise, each separated by 1 min) in novel environment, and were placed back in the startle-paired environment the next day (Fig. 2d). Morphine-treated animals did not differ from controls with respect to baseline motion (Fig. 2e. *t*_25_ = 0.46, *p* = 0.65) or startle induced increases in motion (Fig. 2e. *t*_25_ = 0.19, *p* = 0.85). Moreover, morphine-treated animals did not differ with respect to baseline freezing when initially placed in the startle-paired environment (Fig. 2f. *t*_25_ = 0.11, *p* = 0.91). However, morphine-treated animals did show increased post-startle freezing (Fig. 2f. *F*_1,25_ = 5.17, *p* = 0.03). Importantly, this within-session freezing is a learned behavior [19]. Nevertheless, when placed back into the startle-paired environment the next day, morphine-treated animals did not significantly differ from saline-treated animals (Fig. 2f. *t*_25_ = 1.23, *p* = 0.23).

Taken together, these results strongly suggest that changes in SEFL are independent of changes in general anxiety or stimulus sensitivity. Instead, they suggest that opioid treatment influences the ability of trauma to alter aversive learning.

### Chronic opioid treatment potentiates trauma-induced changes in fear learning and not fear expression

**Fig. 3:**
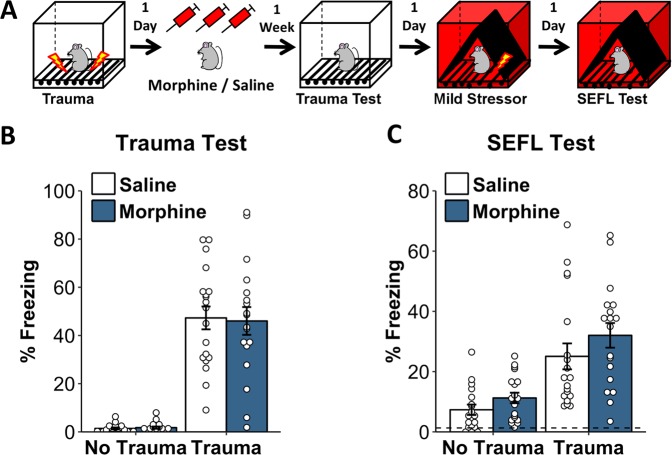
Chronic opioid pretreatment potentiates trauma-induced changes in fear learning and not fear expression. **a** Experiment schematic. Chronic morphine and a week of drug cessation were given in-between the trauma and the rest of the SEFL procedure. **b** Morphine-treated animals do not display altered fear of the trauma context when placed back into it, demonstrating that morphine administration does not increase fear expression. **c** Morphine-treated animals display a trend toward heightened fear in the SEFL test, but this did not reach significance. Dashed line in **c** reflects average pre-shock baseline freezing on the previous day. Error bars reflect standard error of the mean. *N* = 18–19/group

To address whether opioid pre-exposure alters the induction or expression of enhanced fear, we examined the impact of giving chronic opioid treatment after the initial trauma but before the rest of the SEFL procedure (Fig. 3). Because this was the first time a fear memory acquired prior to morphine administration was examined subsequent to it, this experiment allowed us to assess whether morphine acted merely to impact *fear expression*, or whether it altered *fear learning*. Furthermore, because the trauma was experienced before morphine exposure, we were able to further explore the nature of the interaction between trauma and opioid exposure in impacting subsequent fear learning. Upon being returned to the trauma environment after morphine treatment, animals given morphine did not display altered fear of the trauma environment (Fig. 3b. Effect of morphine: *F*_1,70_ = 0.02, *p* = 0.89. Morphine × trauma interaction: *F*_1,70_ = 0.04, *p* = 0.84). Thus, morphine exposure did not enhance the expression of a previously acquired fear memory. Moreover, the ability of morphine exposure to augment subsequent fear learning about a mild stressor was no longer apparent (Fig. 3b. Effect of morphine: *F*_1,70_ = 2.87, *p* = 0.1. Morphine × trauma interaction: *F*_1,70_ = 0.23, *p* = 0.63). This suggests that the ability of morphine to augment SEFL depends upon it influencing changes produced by trauma.

### Chronic opioid pretreatment potentiates SEFL independent of treatment frequency and bouts of acute withdrawal

**Fig. 4:**
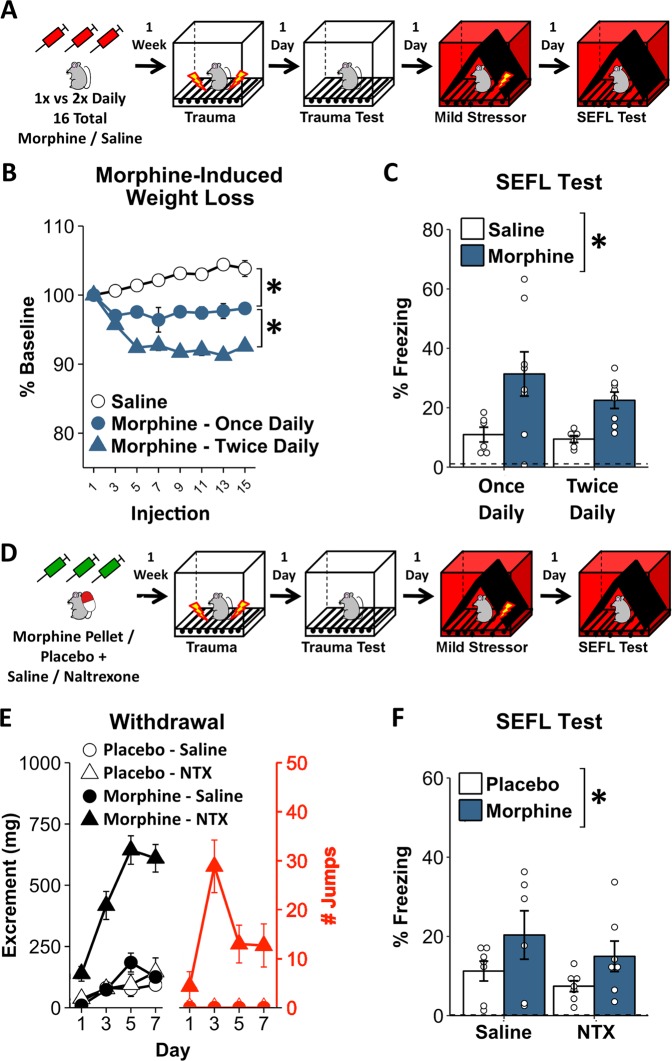
Chronic opioid pretreatment potentiates SEFL independent of acute withdrawal. **a** To manipulate opioid treatment frequency, animals were given 16 saline/morphine injections, either over 8 (2× daily) or 16 days (1× daily), and a week later were run through the SEFL procedure. **b** Twice-daily morphine produces more drastic weight loss than once-daily morphine. **c** Irrespective of injection frequency, morphine treatment potentiated SEFL. **d** To manipulate opioid withdrawal, animals were implanted with placebo/morphine pellets, providing a sustained release of morphine, and were then given twice-daily injections of saline/naltrexone twice/day for 7 days to precipitate withdrawal. A week after the last injection, at a time when morphine is fully out of their system, all animals were run through the SEFL procedure. **e** Morphine animals treated with naltrexone display robust withdrawal. **f** Irrespective of the amount of withdrawal, animals implanted with morphine pellets display heightened SEFL. Dashed line in **c**/**f** reflects average pre-shock baseline freezing of all animals on the previous day. Error bars reflect standard error of the mean. Asterisk reflects significance at *p* < 0.05. Asterisks next to legend denote main effect of morphine treatment. NTX naltrexone. For **b**, **c***:* *n* = 6–8/group. For **e**, **f**: *n* = 6–7/group

The experience of acute withdrawal is frequently posited to underlie the changes in negative affective states associated with addiction [20, 21]. In order to assess the relative contribution of negative affective states produced by acute withdrawal on the observed enhancement in fear learning we separately manipulated the frequency of opioid exposure, which alters the impact of opioid treatment, and the frequency and magnitude of acute withdrawal (Fig. 4).

To manipulate opioid treatment frequency, animals were given 16 saline/morphine injections, but either over 8 days (2× daily) or 16 days (1× daily; Fig. 4a). Demonstrating that this manipulation altered the impact of opioid exposure, mice treated with morphine once per day lost substantially less weight than mice treated twice per day: by the end of morphine administration, animals treated twice per day had lost more weight than those treated once per day (Fig. 4b. *t*_14_ = 4.28, *p* < 0.001), although animals treated with morphine once per day had lost some weight relative to saline-treated animals (Fig. 4b. *t*_18_ = 3.95, *p* < 0.001). However, regardless of the frequency of morphine administration, morphine-treated animals showed a similar enhancement in SEFL (Fig. 4c. Effect of morphine: *F*_1,24_ = 12.27, *p* < 0.01; effect of frequency: *F*_1,24_ = 1.18, *p* = 0.29; morphine ×  frequency interaction: *F*_1,24_ = 0.59, *p* = 0.45).

To provide a more drastic manipulation of acute withdrawal, we implanted mice with subcutaneous morphine pellets, which have been shown to provide a continual release of morphine that drops off slowly over 5–7 days [22, 23]. This allowed us to prevent mice from cycling through repeated acute withdrawal. Then, in order to experimentally mimic the repeated acute withdrawal process, half of the animals received twice-daily injections of the opioid antagonist naltrexone (0.25 mg/kg, i.p.) for 7 days, which precipitates withdrawal (Fig. 4d). To confirm withdrawal behaviors across the course of naltrexone treatment, we examined defecation and jumping behavior, two classic metrics of withdrawal [23]. As can be seen in Fig. 4e, only morphine animals injected with naltrexone displayed increases in these behaviors (morphine × naltrexone interaction on defecation: *F*_1,23_ = 56.03, *p* < 0.001; effect of naltrexone on defecation in morphine group: *F*_1,11_ = 127, *p* < 0.001; effect of naltrexone on defecation in placebo group: *F*_1,12_ = 0.38, *p* = 0.55; Only morphine-naltrexone animals showed any jumping behavior). A week after the last naltrexone injection, at a time in which morphine has been out of the animals’ system for several days [23], animals underwent the SEFL procedure. Supporting a lack of analgesic effect at this timepoint, morphine-treated animals displayed no differences in locomotor response to shock at the time of trauma (effect of morphine: *F*_1,23_ = 1.05, *p* = 0.32; morphine × naltrexone interaction: *F*_1,23_ = 0.51, *p* = 0.48. Data not shown). Despite the enormous differences in withdrawal behaviors, in the final SEFL test animals implanted with morphine pellets expressed enhanced freezing relative to animals implanted with placebo pellets, and there was no impact of naltrexone treatment (Fig. 4f: effect of morphine: *F*_1,23_ = 5.13, *p* = 0.03; effect of naltrexone: *F*_1,23_ = 1.56, *p* = 0.22; morphine × naltrexone interaction: *F*_1,23_ = 0.05, *p* = 0.83). Consequently, acute morphine withdrawal is unlikely to have produced the effects we observed on fear learning.

### Neural correlates of enhanced fear learning following opioid exposure

**Fig. 5:**
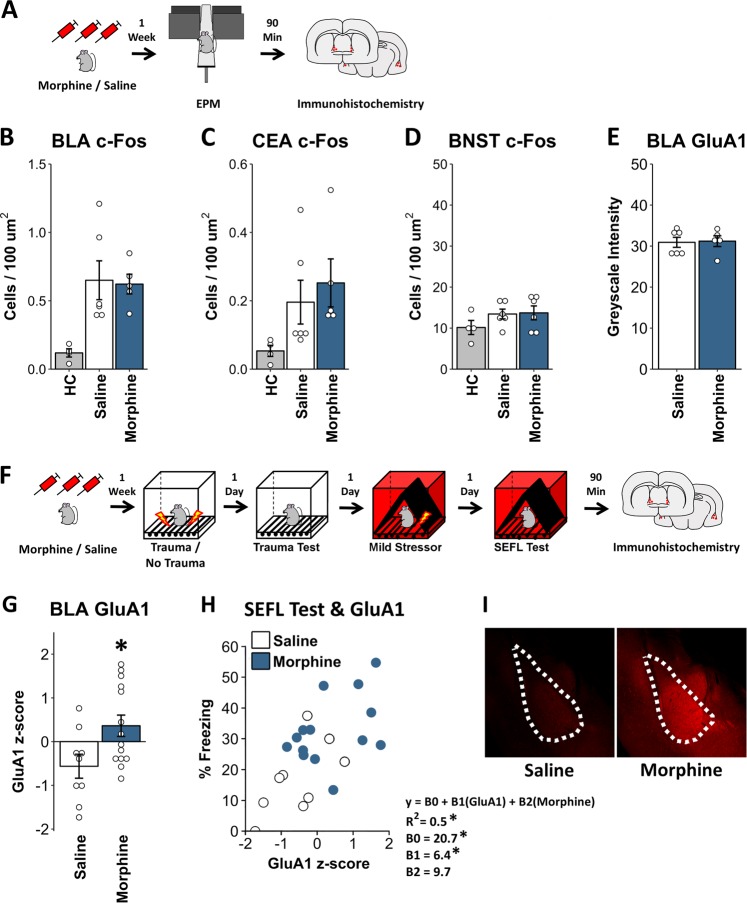
Biological correlates of enhanced fear learning following opioid exposure. **a**–**e** Post-EPM induced c-Fos and GluA1 across anxiety circuitry. **a** Experiment Schematic. In order to examine basal levels of activity across fear and anxiety circuitry, morphine/saline-treated animals were tested in the EPM after a week of cessation and tissue was taken for c-Fos and GluA1 immunohistochemistry. Homecage controls (HC) were treated with saline/morphine but were not placed in the EPM. No differences in induced c-Fos protein were observed in the BLA (**b**), CEA (**c**), or BNST (**d**) between morphine- and saline-treated mice. See Fig. S2 for fos images. **e** Additionally, no differences in GluA1 immunofluorescence were found in morphine-treated mice at this timepoint. **f**–**e** Post-learning changes in BLA GluA1 in morphine-treated mice. **f** Experiment schematic. In a set of animals treated with saline/morphine and run through the SEFL procedure, tissue was taken after the final SEFL test and GluA1 immunohistochemistry was performed in the BLA. **g** Morphine-treated animals displayed greater GluA1 immunofluorescence in the BLA after SEFL than saline-treated animals and **h** regression analysis predicting SEFL test freezing from GluA1 levels and morphine treatment showed that levels of GluA1 were predictive of SEFL, over and above the effect of morphine treatment. Regression equation and coefficients are adjacent to scatterplot. **i** Exemplary images of GluA1 expression in the BLA of saline and morphine animals. Error bars reflect standard error of the mean. Group Sizes for **a**–**e**: homecage: *n* = 4 (two saline, two morphine); saline: *n* = 6; morphine: *n* = 6. For GluA1, only non-Homecage animals were assessed. Group sizes for **f**–**i**: 9–14/group

Having demonstrated that chronic opioid pre-exposure is able to alter the ability of trauma to sensitize fear learning in a withdrawal-independent manner, we next sought to identify functional differences in brain regions associated with anxiety and fear learning which may support this change. A set of animals were treated with the morphine regimen previously described and were then then exposed to the EPM prior to collecting tissue for immediate early gene immunohistochemistry (Fig. 5a–e). This was done in order to examine baseline levels of neural activity that might predate the enhanced SEFL phenotype. Here, prior morphine exposure did not alter c-Fos protein levels in multiple regions critical to fear learning and anxiety (basolateral amygdala, BLA; central nucleus of the amygdala, CEA; and bed nuclei of the stria terminalis, BNST). Although c-Fos was induced in the BLA and CEA in response to EPM exposure (orthogonal contrasts of experimental animals vs. homecage controls: BLA: *t*_12_ = 3.61, *p* < 0.01; CEA: *t*_12_ = 2.14, p = 0.05; BNST: *F*_12_ = 1.49, *p* = 0.16), there were no differences in EPM-induced c-Fos between saline- and morphine-treated animals in these regions (BLA: *t*_12_ = 0.19, *p* = 0.85; CEA: *t*_12_ = 0.68, *p* = 0.57). Additionally, no differences in GluA1 immunofluorescence were found in morphine-treated animals within the BLA (*t*_9_ = 0.16, *p* = 0.88).

Because the SEFL trauma increases GluA1 AMPA receptor subunit protein levels in the BLA [11]—an endpoint of long-term potentiation necessary for fear learning to occur [24]—we lastly assessed BLA GluA1 protein levels in morphine-treated animals that had undergone the SEFL procedure (Fig. 5f–h). We found that post-learning enhancements in GluA1 expression were greater in morphine-treated animals (Fig. 5g. *t*_21_ = 2.46, *p* = 0.02). Moreover, GluA1 expression was predictive of the enhancement in SEFL (Fig. 5h). Multiple regression analysis jointly predicting SEFL test freezing from GluA1 protein levels and morphine treatment (*y* = *B*0 + *B*1×GluA1 + B2×morphine) revealed that GluA1 was a significant predictor of SEFL test freezing (Fig. 1f. *B*1 = 6.36, *t* = 2.56, *p* = 0.02), above and beyond the influence of morphine (*B*2 = 9.67, *t* = 1.99, *p* = 0.06). Thus, while morphine alone did not change GluA1 protein levels, it potentiated the increase in GluA1 triggered by stress, supporting the idea that plasticity within fear learning-circuitry is enhanced in morphine-treated animals.

## Discussion

Here we demonstrate a dramatic ability of chronic opioid regimens to subsequently potentiate SEFL, a finding that may provide insight into the comorbidity between PTSD and opioid dependence. Given that this potentiation lasts for some time beyond discontinuation of drug exposure, it is possible that sensitization of fear learning predisposes individuals who have used opioids—either as prescribed or illicitly—to develop PTSD.

It is important to emphasize that the observed enhancement in fear appears to be a consequence of a facilitation of the biological processes that give rise to the formation of fear memories, as opposed to some other cognitive or physiological process that influences fear expression. First, great care was taken to ensure that the differences observed in opioid-treated animals were not the consequence of increased sensitivity to shock, the aversive stimulus used in the bulk of these experiments. Across a wide range of shock amplitudes, we were unable to detect differences in shock-induced motor responses between saline- and morphine-treated animals, despite being within the dynamic range to detect such differences. Although it could be argued that the motor responses measured do not parallel the internal subjective response to shock (a problem inherent in most animal pain assays because they rely upon a behavioral response), this is unlikely to be the case. After repeated trials, maximal freezing to a stimulus paired with a shock is directly related to shock magnitude [25, 26]. However, we found no differences in freezing between morphine- and saline-treated animals at the trauma test, when freezing is at asymptotic levels, further suggesting that the perceived magnitude or aversiveness of the stimulus was not different. Moreover, we found increased freezing to auditory startle stimuli in morphine-treated animals, suggesting that enhancements in aversive learning were not exclusive to painful shocks.

The increase in fear learning observed is also unlikely to be a consequence of a general state of increased anxiety following opioid treatment. Morphine-treated animals did not display altered fear generalization following trauma when they were placed in a novel context prior to receiving the mild stressor, nor did they differ in their exploration of the open arms of an EPM at a time in which they show heightened fear learning. This suggests that they were not more anxious. Moreover, when animals experienced a trauma prior to morphine exposure, animals did not differ with respect to freezing when subsequently returned to the context of the traumatic event, providing evidence that expression of a previously learned fear response is unaffected. Consequently, morphine appears to have altered the ability with which negative experiences affect future behavior—that is to say, learning.

Chronic opioid exposure prior to trauma, but not after trauma, facilitated SEFL. This finding suggests that the biological changes induced by chronic opioid treatment must be present at the time of trauma for fear learning to be enhanced by morphine. In other words, chronic opioid exposure alters the induction of SEFL. There are several points worth mentioning about this finding. First, in the experiment where morphine was administered after trauma, saline-treated animals appeared to show a greater SEFL effect than when saline/morphine was administered before trauma. This is potentially due to the longer delay between the traumatic experience and the mild stressor in this experiment. Although we have not formally tested this possibility, perhaps SEFL is subject to incubation. Nevertheless, because saline-treated animals still froze less than 30% in the final SEFL test, it is hard to argue that a ceiling effect prevented our ability to detect an increase in morphine-treated animals. It is also noteworthy that when morphine was given following trauma there was a trend for morphine to augment fear in the final SEFL test (*p* = 0.1), although this took the form of a main effect and not an interaction with traumatic experience. In combination with the finding that morphine potentiated freezing to startle stimuli, despite these animals only having experienced a mild stressor previously, it appears that the ability of opioid exposure to augment fear learning is not entirely dependent upon traumatic experience. However, it is substantially clearer when the two are combined.

Opioid pre-exposure, and not one of its major sequelae, acute withdrawal, appears to be directly responsible for this change in stress reactivity. Repeated and robust withdrawal did not augment the ability of opioid pretreatment to enhance SEFL. Moreover, reducing the frequency of morphine administration to weaken its impact similarly did not mitigate enhancements in SEFL. These findings indicate that the enhancement in SEFL by morphine is not a consequence of the stress of repeated morphine withdrawal, but instead might be a consequence of alterations in circuitry associated with morphine pre-exposure. It is worth mentioning that despite manipulating withdrawal, these experiments did not directly compare the amount of dependence induced by various morphine procedures. It is conceivable that the critical variable is whether or not dependence is produced, irrespective of observable withdrawal symptoms. For instance, it has previously been reported that long access to heroin self-administration produces greater levels of dependence, anxiety, and drug seeking than short access heroin self-administration in rodents [27, 28], and in humans it has been shown that prior opioid use is associated with the development of PTSD and treatment-resistant depression [29–31]. Whether these changes are a consequence of dependence, acute withdrawal, cumulative opioid exposure, or comorbid pathology has not been disentangled. Regardless, because dependence is often unavoidable in the medicinal use of opioids, even when withdrawal can be minimized, the finding that changing the amount of withdrawal experienced does not change the observed potentiation of fear learning is of clear clinical interest.

In conjunction with the finding that morphine exposure enhances SEFL, an altered marker of synaptic plasticity within the BLA was also found in morphine-treated animals. Chronic morphine treatment resulted in post-learning, but not pre-learning, increases in the GluA1 subunit of the AMPA receptor within the BLA. Moreover, the degree of GluA1 increase was found to be highly predictive of the amount of SEFL displayed. Importantly, the BLA is thought to be the site of synaptic plasticity supporting associative fear learning [32, 33], production and insertion of GluA1-containing AMPA receptors are a correlate of long-term potentiation (LTP) [34, 35], and blocking incorporation of these receptors into the membrane within the amygdala has been shown to block associative fear learning [24]. Thus, in addition to the behavioral indicators that fear learning is enhanced, we also find that a component of the molecular cascade that supports fear learning was similarly enhanced. Unfortunately, it is currently not possible to directly test the causal contribution of this GluA1 upregulation to the enhancement in SEFL seen following opioid exposure. This is because any manipulation that would reduce the GluA1 upregulation in morphine-treated animals is also anticipated to produce drastic impairments in fear learning in morphine-naïve mice, as has been previously shown [24]. As such, it would be difficult to conclude anything specific about a morphine-induced enhancement from this sort of manipulation. Therefore, here we simply take enhanced GluA1 expression in the BLA as corroborative evidence that BLA-dependent learning is changed by morphine. Future work hopes to isolate the molecular cascade leading to this change. For instance, given that opioid receptors are located throughout the fear circuitry [36, 37], how might chronic opioid treatment act on specific receptors to alter the functional dynamics of this circuitry? Mu opioid receptors are richly expressed within the BLA, although given a general paucity of mu mRNA within the BLA, these receptors are likely to be located on presynaptic afferents [36, 37]. Perhaps chronic opioid exposure and subsequent receptor desensitization/internalization opposes the inhibitory influence these receptors normally have on excitatory inputs to the BLA, which could in turn facilitate fear. Alternatively, kappa opioid receptors are also expressed within the amygdala and have been shown to regulate anxiety [38, 39]. It is conceivable that chronic activation of kappa receptors potentiates fear learning. By understanding the receptor signaling pathways leading to this change, it may be possible to mitigate the impact of opioids on fear.

In closing, these results provide compelling evidence that chronic opioid exposure is able to robustly enhance the ability of trauma to sensitize fear learning. Given the striking comorbidity between PTSD and opioid dependence, as well as the growing prevalence of opioid use and dependence in our society, these findings provide a potential mechanistic link between these conditions and further call into question the safety of both licit and illicit opioid use.

## Funding and disclosure

This work was supported by NIDA DA005010, NIMH RO1 MH115678, NIMH F31 MH108257-02, and NIDA 5T32DA007135. MSF serves as director of research for Neurovation Labs. ZTP, JMT, AKR, KL, WMW and CE have no financial disclosures to report.

## Supplementary information

Supplemental Material
