# Supplementary material for: Chronic opioid pretreatment potentiates the sensitization of fear learning by trauma
Source: Neuropsychopharmacology. 2019 Dec 2;45(3):482–90. doi: 10.1038/s41386-019-0559-5 (PMC6968993; doi:10.1038/s41386-019-0559-5)
Supplement: Supplementary file 1 — Supplemental Material [file 41386_2019_559_MOESM1_ESM.docx]

**Supplementary Figures and Methods**

***Figure S1*: Morphine-induced enhancements in SEFL are observed in both sexes**. A-B) Female and male mice display similar weight changes in response to morphine. C-D) Female and male mice show similar responses to trauma. D-E) Female and male mice show a similar potentiation of SEFL by morphine. At no point did we detect a sex x drug interaction on behavior (all p’s > 0.05). Error bars reflect standard error of the mean. Total n=93: Saline=46, Morphine=47, Females=32, Males=61.


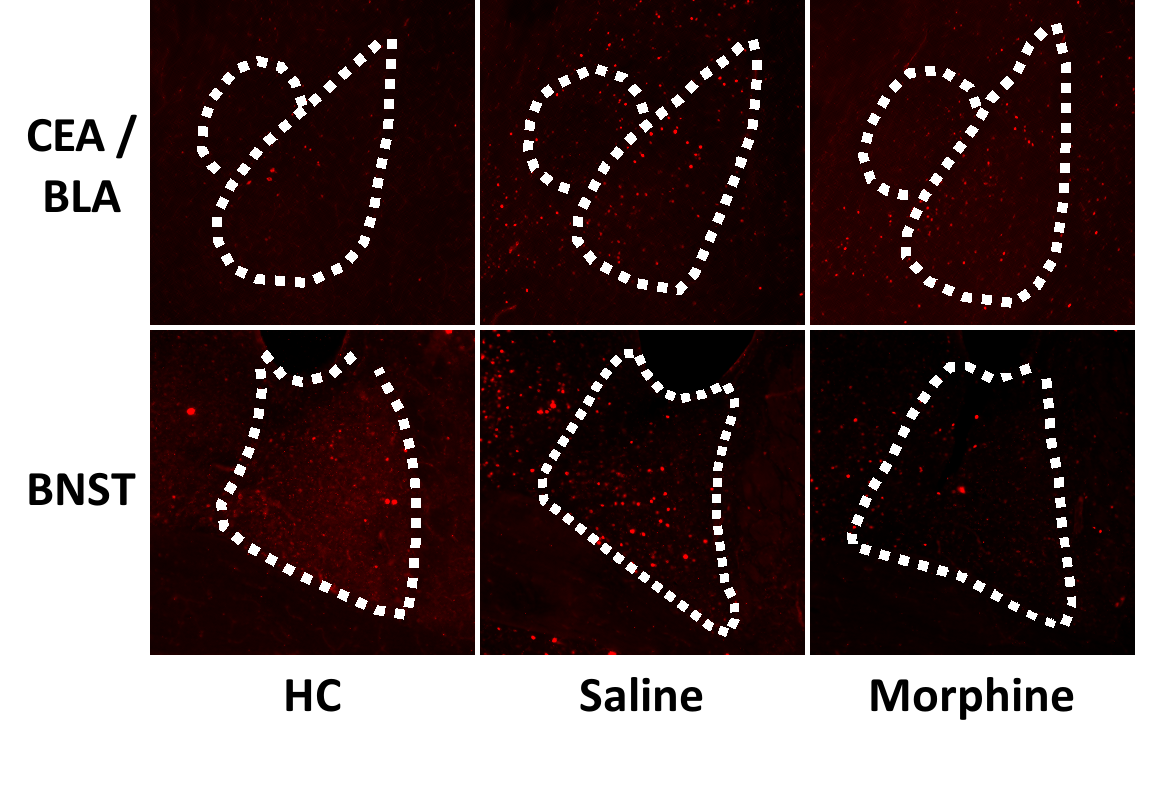


***Figure S2:* EPM-Induced c-fos in saline- and morphine-treated animals.** Exemplary c-fos images from animals chronically treated with saline or morphine and then either left in the homecage (HC) or exposed to the EPM (Figure 5A-E in main text). Images were cropped and resized for publication but are otherwise unaltered. CEA=amygdala, central nucleus; BLA=amygdala, basolateral nucleus; BNST=bed nucleus of the stria terminalis (dorsal component).

**Supplemental Methods:**

*Fear Conditioning Apparatus:*

All fear conditioning procedures took place in Med Associates conditioning chambers (VFC-008; 30.5 x 24.1 x 21 cm), controlled by Med Associates Video Freeze software (Med Associates, St. Albans VT). Chambers were configured to represent distinct environments, differing in physical appearance, luminosity, odor, and background noise. Transport to the different contexts was also varied to aid in discriminability: animals were transported to one context in their home cage and to the other in a separate opaque box with sawdust bedding. Scrambled shocks were delivered to grid floors in the chambers via Med Associates shock scramblers (ENV 414-S). Sessions were recorded by near infrared cameras and freezing and motion were measured using Med Associates Video Freeze software. Using this software, motion was calculated as the average number of pixels whose greyscale value changed per frame (30 frames/second) during a specified time. Freezing was defined as motion below a threshold that conformed to visual inspection of behavior, lasting at least 1 second (1-6).

*Elevated Plus Maze Test:*

The elevated plus maze (EPM) had 4 intersecting arms, each measuring 29.2 x 7.6 cm, suspended 53.3 cm above the floor. The two opposing enclosed arms had opaque walls that were 14.5 cm tall along their length. The elevated plus maze was located in a well-lit room. Behavior was recorded by a camera suspended above the maze. Time spent in the open/closed arms and distance travelled was assessed using EthoVision (Noldus, Wageningen, The Netherlands). Following 2 days of transport habituation to a room adjacent to where the elevated plus maze was located, animals were tested by being placed in the central portion of the maze and allowed to freely explore for 5 min. Percent of time in the open arms was measured, as well as the number of discrete entries into the open arms.

*Shock Sensitivity Test:*

In order to obtain a parametric assessment of shock sensitivity, animals were exposed to shocks of increasing intensity during a single session. Each shock was 2 sec long, and 1 min separated successive shocks. Shocks ranged from 0.1-0.5 mA, in 0.05 mA increments. Each shock intensity was repeated twice, in succession. Motion during the shocks as well as during an equivalent pre-shock period were measured.

*Tissue Collection, Immunohistochemistry and Cell Counts:*

For immunohistochemical staining, brains were rapidly extracted 90 minutes after behavior and placed in cold 4% paraformaldehyde overnight before being transferred to 30% sucrose in 1X PBS. Once brains had sunk, tissue was frozen at -80 °C prior to being sectioned at 40 microns and collected in PBS.

For c-fos staining, tissue was first incubated overnight at 4 °C in a blocking solution (3% normal goat serum, 0.3% Triton X-100, 1X PBS) containing a polyclonal rabbit anti c-fos antibody (1:10,000; Millipore: ABE457). Tissue was then washed 3x in 1X PBS prior to being incubated in blocking solution containing goat anti-rabbit Alexa 594 antibody for 2-4 hours (1:500; Thermo Fisher Scientific: A-11012). Tissue was then washed in PBS, mounted on slides, and coverslipped with Vectashield with DAPI (Vector Laboratories, Burlingame CA).

GluA1 staining was performed using a nearly identical protocol to c-fos except for the primary antibody (Abcam Rabbit Anti Glutamate Receptor 1: AB31232. 1:1000 dilution). Additionally, a 45-minute blocking step preceded incubation in primary antibody.

Multi-channel images were taken using a Keyence BZ-X710 fluorescent microscope at 4x-10x magnification (10x for BNST only). All images within a brain region/experiment were captured using identical microscope/camera settings.

After c-fos staining and image acquisition, images were subsequently processed using an automated cell counting procedure developed in-house using Image J software (imagej.nih.gov). In brief, after converting images to greyscale, a background subtraction procedure using a rolling ball radius equal to the maximum radius of any cell observed was performed to account for differences in background fluorescence between images. Subsequently, images were thresholded and a watershed procedure was used to separate adjoining cells. Cells within a region of interest were then counted utilizing Image J’s particle analysis function with minimum/maximum particle size criteria to exclude artifacts. All parameters were calibrated to a sample of manually counted images such that the automated procedure yielded >90% congruent results in cell counts/location. The same parameters were applied to all images. Regions of interest were traced manually from DAPI channel images to reduce bias.

For GluA1 image analysis, after greyscale conversion, fluorescent intensity was calculated within the region of interest for several images for each animal. The average intensity across images was then calculated, weighted by the area of the region of interest in each image. When animals from different batches of immunohistochemistry were combined (max=2 batches), average intensity values from each batch were first z-scored separately and z-scores were then analyzed.

Images of the central nucleus of the amygdala (CEA) and BLA were taken between -1.2 and -2.2 mm relative to bregma, according to the atlas of Franklin and Paxinos (7). Per animal, 8 to 20 images of each region were taken and counts were normalized to the cumulative surface area of the region of interest across images. Images of the BNST were taken between 0.38 to -0.1 relative to bregma, focusing on the dorsal BNST. Per animal, 6-12 images were taken.

*Statistical Analysis:*

For multifactorial designs, omnibus ANOVA were initially performed, followed by analysis of simple interactions when higher order interactions were present, followed by Bonferroni-corrected post-hoc comparisons. For unifactorial ANOVA, orthogonal contrasts were used when groups could be segregated in an *a priori* manner. For repeated measures ANOVA, when sphericity was violated, the Greenhouse-Geisser correction was used. Nevertheless, unadjusted degrees of freedom are presented for ease of identifying group sizes; p values reflect correction for sphericity. Multiple regression was used to predict freezing from GluA1 z-scores and morphine treatment group membership, dummy-coded such that Saline=0, Morphine=1.

References and Literature Cited

1. Perusini JN, Meyer EM, Long VA, Rau V, Nocera N, Avershal J, et al. (2016): Induction and Expression of Fear Sensitization Caused by Acute Traumatic Stress. Neuropsychopharmacology. 41:45-57.

2. Poulos AM, Reger M, Mehta N, Zhuravka I, Sterlace SS, Gannam C, et al. (2014): Amnesia for early life stress does not preclude the adult development of posttraumatic stress disorder symptoms in rats. Biol Psychiatry. 76:306-314.

3. Poulos AM, Zhuravka I, Long V, Gannam C, Fanselow M (2015): Sensitization of fear learning to mild unconditional stimuli in male and female rats. Behav Neurosci. 129:62-67.

4. Zelikowsky M, Bissiere S, Fanselow MS (2012): Contextual fear memories formed in the absence of the dorsal hippocampus decay across time. J Neurosci. 32:3393-3397.

5. Zelikowsky M, Hast TA, Bennett RZ, Merjanian M, Nocera NA, Ponnusamy R, et al. (2013): Cholinergic blockade frees fear extinction from its contextual dependency. Biol Psychiatry. 73:345-352.

6. Zelikowsky M, Bissiere S, Hast TA, Bennett RZ, Abdipranoto A, Vissel B, et al. (2013): Prefrontal microcircuit underlies contextual learning after hippocampal loss. Proc Natl Acad Sci U S A. 110:9938-9943.

7. Franklin KBJ, Paxinos G (2008): The mouse brain in stereotaxic coordinates. 3 ed. New York, NY: Elsevier Inc.
